# Clinical acupuncture therapy for chronic cholecystitis: A protocol for systematic review and meta-analysis

**DOI:** 10.1097/MD.0000000000024994

**Published:** 2021-03-12

**Authors:** Genping Zhong, Yinghua Luo, Zhenhai Chi, Yunxiu Zhang, Wei Xu, DaoCheng Zhu, Jun Li, Xingyao Hu, Lin Jiao

**Affiliations:** a The Affiliated Hospital of Jiangxi University of Traditional Chinese Medicine, Nanchang; b Central Health Center of Juncun Township, Xingguo County; c Jiangxi University of Traditional Chinese Medicine, Nanchang, China.

**Keywords:** acupuncture, chronic cholecystitis, protocol, systematic review and meta-analysis, therapy

## Abstract

Chronic cholecystitis is a common chronic disease in clinical practice. The incidence of chronic cholecystitis is gradually increasing due to changes in eating habits and even if acute infections aren’t treated in time, it can cause serious complications, continue to plague people's daily life and become an economic burden to society. Currently, the curative effect of chronic cholecystitis under the control of western medicine is still lacking and there are adverse reactions. However, based on current clinical controlled trials acupuncture therapy for chronic cholecystitis has gradually become a complementary treatment. Therefore, this systematic review aims to explore the safety and feasibility of acupuncture therapy in the treatment of chronic cholecystitis.

**Methods:**

We will search the following databases: Medline, PubMed, Cochrane Database of Systematic Reviews, Embase, Chinese Biomedical Literatures Database, China National Knowledge Infrastructure, Wang Fang Database, Chinese Scientific Journal Database from inception to February 2021 without any language restriction. At the same time, relevant literature will be searched manually. The main search terms include: “Acupuncture,” “Cholecystitis.” Data entry will be completed by 2 researchers separately. After entry, cross-checking will be performed to ensure the authenticity of the information. The main outcome criteria include: including the total effective rate of the patient; the traditional Chinese medicine symptom score of the patient includes: abdominal pain, tenderness in the right upper abdomen, and so on; secondary outcome criteria include: gallbladder contraction function and gallbladder thickness, VAS scores, recurrence rate, adverse reactions; use Cochrane risk bias assessment to evaluate and score the included randomized controlled trial; meta-analysis will be performed using RevMan 5.4.0 software. The heterogeneity test is based on the thresholds of *P* and *I*^2^, In order to use solid or random effects models.

**Results:**

This systematic review only evaluates the safety and limitations of acupuncture therapy in the treatment of chronic cholecystitis. We will report the full text in the near future.

**Conclusion:**

This study will explore the safety and limitations of acupuncture therapy in the treatment of chronic cholecystitis, so that acupuncture therapy will be more widely used clinically.

**Trial registration number:**

INPLASY202120020

## 1 Background

### 1.1 Introduction

Chronic cholecystitis is a chronic persistent disease that can be caused by bacterial infection, cholestasis, improper diet, and other factors. It can cause chronic gallbladder inflammation after repeated attacks of gallbladder stones or acute cholecystitis. The treatment cost of chronic cholecystitis in chronic digestive system diseases are also very high.^[1]^ In clinical patient manifestations, its forms are different, whether there are obvious abdominal symptoms or the main manifestations are repeated right upper abdomen or upper middle abdomen distension or discomfort as the main manifestation and right upper abdomen tenderness or percussion pain, accompanied by abdominal distension Accompanying symptoms of the digestive system such as belching, loss of appetite, and so on.^[2]^ At present, the cause and mechanism of chronic cholecystitis are not completely clear. The cause of the disease can be summarized as 4 major factors: gallbladder stones, bacterial and fungal infections, changes in hemodynamics in the gallbladder, and other factors.^[3,4]^ Its internal mechanism is believed by modern research that chronic cholecystitis is closely related to abnormal bile duct motility and lipid metabolism disorders. Tumor necrosis factor-α (INF-α) is a pro-inflammatory cytokine produced by monocytes and macrophages. It is widely involved in the body's immune response and inflammatory response. Increased tumor necrosis factor-α can cause interleukin 1 and interleukin 6 and other inflammatory factors release, and promote the accumulation of white blood cells in the lesion, aggravate the inflammatory response. The relationship between the level of serum tumor necrosis factor-α and the severity of the disease has been confirmed, which can reflect the degree of inflammation in the body. Leptin is a protein molecule peptide hormone secreted by fat cells. Studies have shown that leptin can participate in inflammation by activating Nuclear factor-κB (NF-κB) that may also participate in the development of chronic cholecystitis by regulating lipid metabolism, so it can be used for evaluation. Disease progression and prognosis.^[5,6]^

Chronic cholecystitis had become one of the digestive diseases that affect people's quality of life and it continues to increase with age. However, recent studies have found that the incidence of chronic cholecystitis in children is gradually increasing at this age.^[7–9]^ At present, the overall fatality rate caused by biliary tract infections has been significantly reduced. The fatality rate has been reduced from more than 50% in the 1970s to less than 10% in the 1980s,^[10]^ but if not treated in time, severe infections will cause systemic inflammation in patients. Response syndrome, sepsis, multiple organ dysfunction, and even death, the fatality rate can be as high as 11% to 27%.^[11]^ The prevalence of chronic cholecystitis in adults is reported in China from 0.78% to 3.91%. The prevalence of gallbladder stones in women is higher than that in men, and the male to female ratio was 1: (1.07–1.69). A large-scale survey of people on physical examinations covering 24 provinces and cities showed that the prevalence of gallbladder stones was 1.1% for people aged 20 to 29 years, the prevalence rate for people aged 30 to 39 was 2.6%, those aged 40 to 49 years was 4.4%, the prevalence rate was 8.0% for 50 to 59 years old, the prevalence rate was 8.3% for 60 to 69 years old, and the prevalence rate was 11.2%.^[12,13]^

Of course, for clinically diagnosed patients with chronic cholecystitis, the clinical manifestations of the patient are still needed. Physical examination and routine abdominal ultrasound examination are the first choice for the diagnosis of chronic cholecystitis and gallbladder stones.^[14]^ For the differentiation of acute cholecystitis and chronic cholecystitis, multidetector computed tomography is the most valuable in the differentiation of adjacent liver enhancement, gallbladder inner diameter increase, wall thickness or increased wall striations, fat opacification or effusion around the gallbladder. Of course, diffusion-weighted imaging of the liver parenchyma around the gallbladder is highly specific for distinguishing acute cholecystitis from chronic cholecystitis.^[15,16]^ If chronic cholecystitis is not properly diagnosed and treated in time, it can cause death. According to statistics, the 30-day all-cause mortality rate is 2.4% to 8.4%. Seriously threaten the lives of patients.^[17]^

In this regard, studies have found that traditional Chinese medicine (TCM) is effective in treating chronic cholecystitis, with few side effects and patients can have a higher quality of life during treatment.^[18]^ Animal studies have found that the treatment of chronic cholecystitis by Chinese medicine can up-regulate the expression of c-Kit to improve gallbladder damage, and improve the contraction response of isolated guinea pig gallbladder muscle strips, thereby alleviating pain.^[19,20]^ Acupuncture is a beneficial complementary therapy. Although it does not directly kill bacteria and viruses that has a certain effect in treating bacterial infections, viral infections, and even sepsis. This clinical fact shows that acupuncture therapy The extensive regulation effect is embodied in the control of excessive release of inflammatory factors, improvement of immune function, protection and protection of multiple organ functions, and so on.^[21]^ Studies had found that electroacupuncture at Yanglingquan point can promote the secretion of cholecystokinin in patients with chronic cholecystitis, slow down the blood flow of the gallbladder artery and increase resistance index. It had also been found that electroacupuncture at Zusanli and Yanglingquan points in rabbits can increase the levels of motilin and cholecystokinin in gastric antrum smooth muscle, Oddi sphincter tissue, and plasma.^[22,23]^ In addition, acupuncture therapy has the characteristics of regulating qi-blood-yin-yang, preventing disease, having less side effects, and being easily accepted by patients. Therefore, acupuncture therapy is popular all over the world.^[24,25]^

The use of systematic analysis is helpful to evaluate the effectiveness and credibility of clinical methods.^[26]^ As discussed in the previous article, the number of patients with chronic cholecystitis is gradually increasing, and the simplicity and low side effects of acupuncture therapy have been recognized by clinicians. Therefore, randomized controlled trials (RCT) using acupuncture therapy for chronic cholecystitis are also increasing. At present, there is still a lack of systematic reviews in clinical practice. Therefore, the safety and effectiveness of acupuncture therapy in the treatment of chronic cholecystitis will be systematically evaluated this time, so that acupuncture therapy can better serve the clinic.

## 2 Methods and analysis

### 2.1 Study registration

The system evaluation plan is registered on the platform, registration number: INPLASY202120020. The authenticity can be checked through the website given in this article (https://inplasy.com/inplasy-2021-2-0020/). There is no need for ethical review this time. It only evaluates the relevant literature in various databases on the treatment of chronic cholecystitis with acupuncture therapy. Manually search for other related studies in the treatment of chronic cholecystitis with acupuncture therapy to find potential applicable research. At the same time, follow evidence-based guidelines for preferred reporting items for systematic reviews and meta-analyses Protocol (the protocol follows the Cochrane Handbook for Systematic Reviews and Meta-Analysis Protocol).

### 2.2 Eligibility criteria

#### 2.2.1 Type of studies

For clinical controlled trials of acupuncture therapy in the treatment of chronic cholecystitis in published Chinese and English literature, manually search for related researches on acupuncture therapy in the treatment of chronic cholecystitis. Of course, non-RCTs must be excluded.

#### 2.2.2 Types of participants

The inclusion of this literature must be RCT that meets the diagnostic criteria for chronic cholecystitis^[27]^ will be included. This article does not limit the age, gender, and source of the patient. Exclude patients with other diseases in patients with chronic cholecystitis.

#### 2.2.3 Types of interventions

Patients with chronic cholecystitis in the test group must use acupuncture therapy as the main program (could be combined with other treatments or used alone), and the control group cannot use acupuncture therapy.

#### 2.2.4 Type of comparators

The control group can include blank control, medicine (TCM, western medicine) treatment, conventional symptomatic treatment, and so on.

(1) Acupuncture therapy versus no treatment;
(2) Acupuncture therapy versus placebo;
(3) Acupuncture therapy versus sham acupuncture;
(4) Acupuncture therapy versus active treatment;

#### 2.2.5 Outcome observation

##### 2.2.5.1 Main outcome indicators

Including the total effective rate of the patient; the TCM symptom score of the patient includes: abdominal pain, tenderness in the right upper abdomen, and so on.

#### 2.2.6 Secondary outcome indicators

(1) Gallbladder contraction function and gallbladder thickness;
(2) VAS score;
(3) Recurrence rate;
(4) Adverse reactions.

### 2.3 Exclusion criteria

If the article is before and after control; the control group of the article uses acupuncture therapy; there are repeated nonrandomized controlled studies such as literature, theoretical review literature, rat, rabbit and other animal experimental research literature.

### 2.4 Search strategy

We will conduct a comprehensive literature search in Medline, PubMed, Cochrane Database of Systematic Reviews (Cochrane Library, Wiley), Embase, Chinese Biomedical Literatures Database, China National Knowledge Infrastructure, Wang Fang Database, Chinese Scientific Journal Database from inception to February 2021 without any language restriction; the main subject terms searched: “Acupuncture ,” “Cholecystitis.” The retrieval strategy of Pubmed database is shown in Table 1. The search strategies of major databases will be adjusted according to the different databases.

**Table 1:** Search Strategy (PubMed).

Order	Strategy
#1	Search “Cholecystitis”[Mesh] OR“Acalculous Cholecystitis”[Mesh]
#2	Search “Chronic Cholecystitis” [Ti/Ab] or “Cholecystitis” [Ti/Ab] or “Acalculous Cholecystitis”[Ti/Ab] or “Emphysematous Cholecystitis” [Ti/Ab] or “Gall Bladder Empyema” [Ti/Ab]
#3	#1 OR #2
#4	Search “Acupuncture”[Mesh] OR “Acupuncture Therapy”[Mesh] OR “Acupuncture, Ear”[Mesh] OR “Acupuncture Points”[Mesh]
#5	Search “acup^∗^” [Ti/Ab] or “need^∗^” [Ti/Ab] or “Acupuncture Therapy”[Ti/Ab] or “Acupuncture treatment ” [Ti/Ab] or “Pharmacopuncture”[Ti/Ab] or “ Acupuncture Points” [Ti/Ab]
#6	#4 AND #5
#7	Search “Randomized controlled trial” [MeSH] or “controlled clinical trial” [MeSH]
#8	Search “Randomized controlled trial” [Ti/Ab] or “clinical trial” [Ti/Ab] or “randomized” [Ti/Ab]
#9	#7 OR #8
#10	#3 AND #6 AND #9

### 2.5 Article screening process

#### 2.5.1 Screening steps

We will process the documents that can be finally used in accordance with certain operating procedures; first, we retrieve all the documents we need from the database according to the correct subject terms, and import them into the document manager using the correct import method In noteExpress 3.0, duplicate documents are deleted in the document manager; then, researchers delete documents that are not related to this systematic review by reading the title and abstract; moreover, download the remaining articles one by one and read the full text; finally, the final document is determined according to the various standards discussed above.

**Figure 1:**
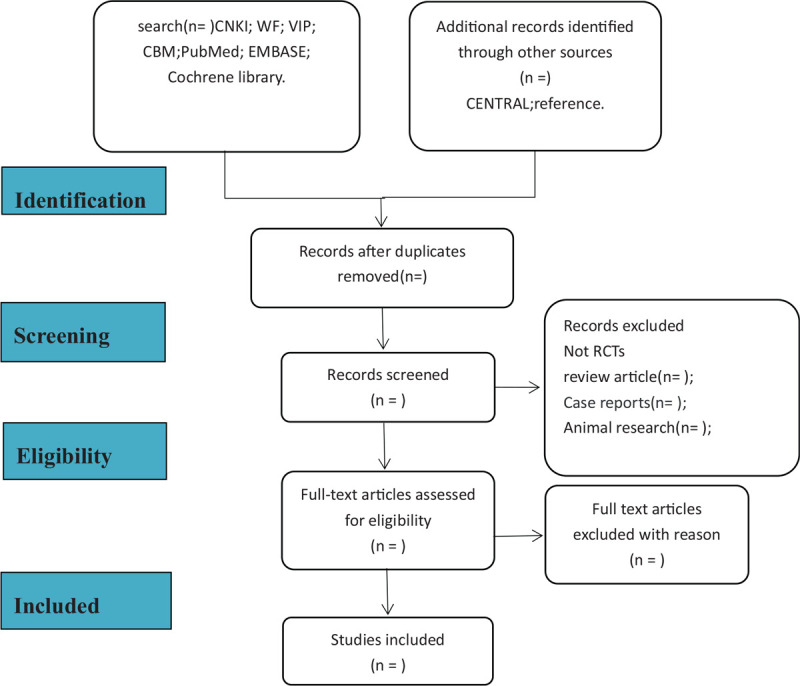
Flowchart of literature selection.

In the included literature, 2 researchers (Wei Xu, Jun Li) strictly followed the operating steps of the systematic review. If they have ambiguities about the same article, they will ask the third reviewer (Genping Zhong) for negotiation. The specific process of the included literature can be seen in Figure 1.

#### 2.5.2 Extraction of literature information

In the process of document inclusion this time, the required inclusion information will be separately extracted by 2 researchers (Wei Xu, Jun Li) and entered into excel 2010 at the same time. When the information extracted by the 2 researchers (Wei Xu, Jun Li) are going to enter, they were cross-checked and checked. If there is any ambiguity in the literature inclusion, you can discuss with a third party (Genping Zhong) to ensure the credibility of the included article. Of course, the included information includes the following content: the author of the article, the title of the document, the sample size, and the outcome index. If the important information that needs to be included in the article is missing, the author of the article will be contacted by phone or email to ensure the authenticity of the information.

#### 2.5.3 Methodological evaluation

The method of article quality evaluation and bias risk assessment will be done through Cochrane Reviewer's Handbook 5.0. The content includes 7 items: random method selection; allocation concealment; blinding method; completeness of the outcome data; whether the rater is blinded; selective reporting of results; other biases, all of the above 7 items contain “yes,” “no,” and “unclear” 3 options. Two evaluators (Wei Xu, Jun Li) should make appropriate evaluations among the options. If there is a dispute between the 2 during the selection process, they can consult a third party (Genping Zhong) for handling.

### 2.6 Data Integration

#### 2.6.1 Data synthesis

Current data can be divided into counting data and measurement data. When there will be technical data in the data, it will be expressed by odds ratio and 95% confidence interval; when there is measurement data, weighted mean difference 95%, indicated by the confidence interval. Use standardized mean difference when there are some units that are not unified.

#### 2.6.2 Describe the heterogeneity of the data

Use *I*^2^ to test the heterogeneity. For the case of using the solid effect model, it will be in line with *P* > .1 and *I*^2^ < 50%; when using the random effect model, it is in line with the case of *P* < .1 and *I*^2^ > 50%. If substantial heterogeneity will be found in the analysis process, descriptive analysis can be used.

#### 2.6.3 Publication bias

The use of Review Manager 5.4 inverted funnel chart to analyze the bias is due to the large number of literatures on acupuncture treatment of chronic cholecystitis. Of course, the funnel chart obtained is for reference only.

#### 2.6.4 Subgroup analysis

When there is a large heterogeneity, the included articles will be analyzed according to different control measures.

#### 2.6.5 Sensitivity analysis

Sensitivity analysis is to evaluate the authenticity of this systematic review and we will use the software STATA 14.0 to perform sensitivity analysis.

## 3 Discussion

This systematic review is the first summary of the safety and effectiveness of acupuncture therapy in the treatment of chronic cholecystitis based on the current modern clinical efficacy research. Its purpose is to find favorable evidence-based evidence for the application of acupuncture therapy to chronic cholecystitis. Based on the discussion part of the article, it will be described in the following aspects:

(1) The pathogenesis of chronic cholecystitis in Chinese medicine;
(2) Research on the advantages and disadvantages of acupuncture therapy in the treatment of chronic cholecystitis and the mechanism of acupuncture and moxibustion;
(3) Horizontal comparison with other treatment methods (Chinese medicine, western medicine, surgery, etc);
(4) Explain the results of this evaluation;
(5) Come to the final conclusion.

Acupuncture therapy has a history of thousands of years of development, and it has been used as a supplement and alternative treatment by doctors at home and abroad.^[28]^ The intervention of acupuncture and moxibustion may act by regulating various pathways and mediators. Therefore, acupuncture therapy has become the dominant disease of chronic cholecystitis.^[29]^ However, the current acupuncture therapy has no systematic scientific evaluation on chronic cholecystitis, so the purpose of this article is to provide acupuncture and moxibustion. The safety and effectiveness of the therapy in the treatment of chronic cholecystitis provide evidence-based medical opinions. Of course, this study still has certain limitations: First, the quality of acupuncture-related RCT literature is generally low, and the trial design is an important reason. TCM emphasizes holistic concepts and treatment based on syndrome differentiation. The treatment measures or programs are often complex, abstract, and comprehensive. The curative effect has the characteristics of multiple effects and multiple approaches and it is a complex intervention with multiple targets. Secondly, the evaluation of the efficacy of Chinese medicine The quality of the cohort study is not high, and the reasons may be various, such as easy to be confused with other clinical trials in design; excessive intervention or control artificially imposed, thereby reducing the external authenticity of the cohort study and deviating from the nature of observational research design; Third, the author of the article may not be contacted, resulting in incomplete data results. Therefore, for more accurate conclusions, more high-quality RCTs and research mechanisms are needed to confirm its effectiveness, so as to more objectively evaluate the safety and effectiveness of acupuncture therapy in the treatment of chronic cholecystitis.

## Author contributions

**Conceptualization:** Genping Zhong.

**Data curation:** Yinghua Luo, Zhenhai Chi.

**Formal analysis:** Genping Zhong, Yunxiu Zhang.

**Investigation:** Yunxiu Zhang, Lin Jiao.

**Methodology:** Jun Li, Xingyao Hu.

**Project administration:** Lin Jiao.

**Resources:** Jun Li.

**Software:** Wei Xu, DaoCheng Zhu, Jun Li.

**Supervision:** Lin Jiao.

**Validation:** Lin Jiao.

**Writing – original draft:** Wei Xu, DaoCheng Zhu, Xingyao Hu, Jun Li.

**Writing – review & editing:** DaoCheng Zhu, Jun Li, Wei Xu, Yinghua Luo, Zhenhai Chi, Yunxiu Zhang, Xingyao Hu.
